# 4457 Adopting a Team Science Communication Module for Community-Partnered Teams

**DOI:** 10.1017/cts.2020.251

**Published:** 2020-07-29

**Authors:** Arleen F Brown, Keith Norris, Rachelle Bross, Yelba Castellon, Norma Mtume, D’Ann Morris, Aziza Lucas Wright, Juan Barron, Sarmen Hakopian, Maritza Salazar Campo

**Affiliations:** 1 UCLA Division of General Internal Medicine; 2 The Lundquist Institute at Harbor-UCLA Medical Center; 3 UCLA General Internal Medicine; 4 Charles Drew University; 5 University of California, Irvine

## Abstract

OBJECTIVES/GOALS: There is increased recognition that patients and community members are critical to creating impactful research. To this end the UCLA CTSI Community Engagement & Research Program modified an established multidisciplinary team science communication module to train academic-community research teams. METHODS/STUDY POPULATION: Community partners who have had previous experience in participatory research provided input such as limiting the emphases of individual academic introductions to group icebreakers (to level the playing field), reduced academic jargon to lay language, reducing the amount of text to key principles, and changed academic team scenarios for the team activity to represent community-academic teams. Academic partners articulated institutional barriers to integrating community into institutional systems. Iterative testing and modifications occurred through pilots with eleven teams (49 individuals). RESULTS/ANTICIPATED RESULTS: Embedding community partners in team science training involved creating a level playing field with less emphasis on academic credentials, using lay language in the didactic sessions and ensuring accessibility in all aspects of the training. An example of modifications: communication scenarios were read out loud by participants, which community partners felt were not inclusive of potential varying literacy levels and all partners may not feel comfortable reading aloud in a group setting. The vignettes were replaced with short videos of the scenarios with audio recordings. Several modifications were made the training’s team activity of the training module. DISCUSSION/SIGNIFICANCE OF IMPACT: Traditional academic team science training required significant modifications for an academic/community-partnered team to allow for optimal collaboration, inclusion, and strategically reduce the power dynamics that can naturally occur. Long-term followup to assess their effectiveness is needed.

